# *Peromyscus leucopus* mouse brain transcriptome response to Powassan virus infection

**DOI:** 10.1007/s13365-017-0596-y

**Published:** 2017-11-16

**Authors:** Luwanika Mlera, Kimberly Meade-White, Eric Dahlstrom, Rachel Baur, Kishore Kanakabandi, Kimmo Virtaneva, Stephen F. Porcella, Marshall E. Bloom

**Affiliations:** 1 Biology of Vector-Borne Viruses Section, Laboratory of Virology, Rocky Mountain Laboratories, NIAID/NIH, Hamilton, MT 59840 USA; 2 Rocky Mountain Veterinary Branch, Rocky Mountain Laboratories, NIAID/NIH, Hamilton, MT 59840 USA; 3 Genomics Unit, Research Technologies Branch, Rocky Mountain Laboratories, NIAID/NIH, Hamilton, MT 59840 USA

**Keywords:** Powassan virus, Tick-borne flavivirus, Peromyscus lecuopus mouse, Brain transcriptome, Differential gene expression, Interferon signaling

## Abstract

Powassan virus (POWV) is a tick-borne *Flavivirus* responsible for life-threatening encephalitis in North America and some regions of Russia. The ticks that have been reported to transmit the virus belong to the *Ixodes* species, and they feed on small-to-medium-sized mammals, such as *Peromyscus leucopus* mice, skunks, and woodchucks. We previously developed a *P. leucopus* mouse model of POWV infection, and the model is characterized by a lack of clinical signs of disease following intraperitoneal or intracranial inoculation. However, intracranial inoculation results in mild subclinical encephalitis from 5 days post infection (dpi), but the encephalitis resolves by 28 dpi. We used RNA sequencing to profile the *P. leucopus* mouse brain transcriptome at different time points after intracranial challenge with POWV. At 24 h post infection, 42 genes were significantly differentially expressed and the number peaked to 232 at 7 dpi before declining to 31 at 28 dpi. Using Ingenuity Pathway Analysis, we determined that the genes that were significantly expressed from 1 to 15 dpi were mainly associated with interferon signaling. As a result, many interferon-stimulated genes (ISGs) were upregulated. Some of the ISGs include an array of *TRIMs* (genes encoding tripartite motif proteins). These results will be useful for the identification of POWV restriction factors.

## Introduction

Powassan virus (POWV) is a neurotropic tick-borne *Flavivirus* (TBFV) responsible for life-threatening meningoencephalitis with necrotizing inflammation and lymphocytic infiltrations in humans (Gholam et al. 1999; McLean and Donohue 1959; Piantadosi et al. 2015). The virus is closely related to deer tick virus (DTV or POWV lineage II), and both belong to the tick-borne encephalitis virus (TBEV) serogroup. POWV was initially described in 1958 in a fatal case involving a 5-year-old boy in the small town of Powassan, Ontario, Canada (McLean and Donohue 1959). Although POWV infections in the USA are sporadic, cases have been reported in several states, such as Connecticut, New York State, New Hampshire, and Massachusetts (Deardorff et al. 2013; Ebel 2010; Hermance and Thangamani 2017; Leonova et al. 2009; Lindsey et al. 2015; Pastula et al. 2016; Piantadosi et al. 2015; Simon et al. 2013). In recent months, additional cases of POWV encephalitis have been reported in the Northeastern United States and some were fatal. POWV is also a significant cause of illness in eastern regions of Russia (Deardorff et al. 2013). The incidence of human cases appears to be on the increase (Deardorff et al. 2013; Ebel 2010; Hinten et al. 2008; Piantadosi et al. 2015). An effective vaccine against the TBEV serogroup is available mainly in Europe, but up to 15,000 new infections continue to be recorded annually, leading to death in close to 40% of infected cases depending on the TBEV strain (Dobler 2010; Heinz and Kunz 2004; Heinz et al. 2013).

In almost all cases, the ticks that have been shown to transmit TBFVs including POWV belong to the *Ixodes* genus. These ticks typically feed on small-to-medium-sized mammals, such as white-footed mice or *Peromyscus leucopus*, striped field mice (*Apodemus agrarius*), skunks (*Mephitis mephitis*), and woodchucks (*Marmota monax*) (Dupuis II et al. 2013; Ebel 2010; Kim et al. 2008; Main et al. 1979; Mlera et al. 2014; Perkins et al. 2003). Evidence implicating these mammals as reservoirs, bridge, or amplification hosts for TBFVs is inconsistent and includes the isolation of the TBEV strain A104 from the brains of wild-caught yellow-necked mice (*Apodemus flavicollis*) in Austria (Frey et al. 2013). The TBEV strains Oshima 08-As and Oshima A-1 were isolated from spleens of captured *Apodemus speciosus*, and the Oshima C-1 strain from the gray-backed vole *Clethrionomys rufocanus* in Japan (Kentaro et al. 2013; Takeda et al. 1999). A report from South Korea describes PCR detection and TBEV isolation from lung and spleen tissue obtained from wild *Apodemus agrarius* mice (Kim et al. 2008). In addition, scientists in Finland detected TBEV RNA in mouse brains, but some mice that had viral RNA were seronegative (Tonteri et al. 2011). Indirect serological evidence suggesting exposure to TBFVs in wild rodents includes the detection of anti-POWV antibodies in wild-caught *Peromyscus truei* and *Peromyscus maniculatus* in New Mexico, *Myodes rutilus* in Siberia and Alaska, and *Myodes gapperi* in Southern Alaska (Deardorff et al. 2013). Recent surveys have also found evidence of antibodies against POWV in 4.2% of Eastern US white tail deer, suggesting that virus-infected ticks might also be feeding on this large mammal (Pedersen et al. 2017). In addition to transmitting virus to the mammalian host, infected ticks can also rapidly infect naïve ticks feeding in proximity via a process called “co-feeding” (Labuda et al. 1993a, 1997, 1993b). Thus, the nature of the interaction between arthropod hosts, potential reservoir species, and virus remains uncertain.

Mice belonging to the *Peromyscus* genus, particularly *P. leucopus* and *P. maniculatus* species, are the most abundant mixed-forest rodents in the USA (Bedford and Hoekstra 2015). However, very little is known about the specific role that these mice play in POWV biology. Our initial effort to decipher this role was the development of a *P. leucopus* model of POWV infection, which is characterized by a lack of overt clinical signs of disease following intraperitoneal or intracranial challenge (Mlera et al. 2017). However, intracranial challenge of *P. leucopus* mice leads to mild subclinical encephalitis at early time points (5 to 15 days post infection (dpi)), but the inflammation resolves by 28 dpi (Mlera et al. 2017). These observations starkly contrasted with inbred laboratory mouse strains C57BL/6 and BALB/c, which succumb to severe and fatal neurological disease upon intracranial inoculation, recapitulating some aspects of human disease (Hermance and Thangamani 2015; Mlera et al. 2017; Santos et al. 2016).

To extend our studies and to characterize the mild encephalitic response in *P. leucopus* mouse brains, we used RNA sequencing (RNA-Seq) to profile the differential transcriptome changes associated with POWV infection. Our results indicate that POWV induces the differential expression of many genes and the *P. leucopus* mice mount a robust interferon response against the virus in a tightly regulated manner. These results will be useful for identification of factors that that have a role in restricting POWV.

## Methods

### *P. leucopus* mouse brain samples

This study was an extension of our previous work in which we intracranially inoculated 4-week-old *P. leucopus* mice with 10^3^ PFU of POWV LB strain, or serum-free DMEM for controls, followed by euthanasia at 1, 3, 5, 7, 15, and 28 dpi (Mlera et al. 2017). At each time point, we infected three mice with POWV, and three mice were mock-infected controls. All mouse work was ethically done in animal biosafety level 3 (BSL3) facilities according to approved animal study protocols, which are stated in a previous report (Mlera et al. 2017). At necropsy, brain tissue samples from POWV-infected mice and controls for deep sequencing were placed into 1 ml of TRIzol, homogenized, and stored at − 80 °C until total RNA extraction.

### Total brain RNA extraction

A 200-μL aliquot of the brain homogenate was combined with 800 μL of TRIzol (Thermo Fisher Scientific, Waltham, MA) and 200 μL 1-bromo-3-chloropropane (Sigma-Aldrich, St. Louis, MO), mixed, and centrifuged at 4 °C at 16,000×*g* for 15 min. The aqueous phase was added to QIAshredder columns (Qiagen, Valencia, CA) and centrifuged at 21,000×*g* for 2 min to fragment any remaining genomic DNA (gDNA) in the sample. The flow-through was combined with an equal volume of RLT buffer (Qiagen, Valencia, CA) with 1% β-mercaptoethanol, and RNA was extracted using AllPrep DNA/RNA 96 Kit as described by the manufacturer (Qiagen, Valencia, CA) including additional on-column DNase 1 treatment to remove gDNA. RNA purity and concentration was determined by spectrophotometry. RNA integrity was analyzed using Agilent 2100 Bioanalyzer (Agilent Technologies, Santa Clara, CA). Equal amounts of RNA (400 ng) were used as a template for NGS library preparation.

### RNA-Seq and data analysis

Host mRNA sequencing used the Illumina TruSeq RNA Sample Preparation Kit v2 (Illumina, San Diego, CA), beginning at the poly-A selection step. Libraries were bar coded, amplification cycles were reduced to 10, and libraries were run as a pool across all eight lanes of the HiSeq 2500 sequencer (Illumina, San Diego, CA).

The generated RNA-Seq reads for each mouse brain were trimmed of adaptor and poor quality sequences and mapped against the *P. maniculatus* genome (assembly Pman_1.0) using TopHat 2 software (Kim et al. 2013). We aligned the RNA-Seq reads to the genome of *P. maniculatus* because a complete *P. leucopus* genome is currently not available. We also mapped the RNA-Seq reads to the genomes of the rat (*Rattus norvegicus* assembly Rnor_6.0), the house mouse (*Mus musculus* assembly GRCm38.p5), and the Chinese hamster (*Cricetulus griseus* assembly CriGri_1.0). Using the Basic Logic Alignment Search Tool (BLAST; https://blast.ncbi.nlm.nih.gov), we compared selected amino acid sequences of *P. leucopus* proteins deposited in GenBank to those of *P. maniculatus*, *Rattus norvegicus*, *Mus musculus*, and *Cricetulus griseus*.

Transcript quantification and differential gene expression analysis was performed for each POWV-infected mouse using the Cufflinks software suite (Trapnell et al. 2013). The triplicate data for each time point were filtered at ± 2-fold change and a false discovery rate (FDR) or corrected *p* value (*q* value) of < 0.05. Filtered data were used to generate pathways, networks, and functional inference using Qiagen’s Ingenuity^®^ Pathway Analysis (IPA^®^) software (Qiagen Redwood City; www.qiagen.com/ingenuity). The sequence data were submitted to the National Center for Biotechnology Information Small Read Archive (http://www.ncbi.nlm.nih.gov/Traces/sra/), BioProject number PRJNA395043.

### qPCR validation of RNA-Seq data

We selected four genes for qRT-PCR validation of RNA-Seq data, and these were *STAT2*, *IFIT3*, *DDX58*, and *TRIM21*. The glycyl-tRNA synthetase gene (*GARS*) was used to normalize the relative expression of the target genes based on CV across the entire sample set, gene function, and expression level. The Express qPCR SuperMix Universal with premixed ROX (Life Technologies, Carlsbad, CA) was used to perform the qRT-PCR assay. The reactions were carried out in 20-μL reactions using forward primer and reverse primers and a fluorescent probe (Table 6). All reactions were performed in triplicate, and no template controls were included in each run. The qRT-PCR reactions were carried out at 50 °C for 2 min, 95 °C for 2 min, 55 cycles of 95 °C for 15 s, and 60 °C for 1 min. Data was analyzed using ABI 7900HT (version 2.4) sequence detection system software (Life Technologies, Carlsbad, CA). The Spearman correlation was calculated using GraphPad Prism 7.01 (GraphPad Prism, La Jolla, CA).

## Results

### Alignment of the *P. leucopus* RNA-Seq reads to reference genomes

A complete *P. leucopus* mouse genome is currently unavailable. Thus, we mapped all the *P. leucopus* sequences generated from our RNA-Seq sequencing to the genomes of *P. maniculatus* (deer mouse), *Rattus norvegicus* (rat), *Mus musculus* (house mouse), and *Cricetulus griseus* (Chinese hamster). Approximately 60% of the *P. leucopus* RNA-Seq reads aligned to the *P. maniculatus* genome, but < 10% of the reads could be mapped to the genomes of the rat, the house mouse, or the Chinese hamster. In addition, GenBank amino acid sequences of *P. leucopus* MAVS, IFNAR1, STAT1, TRIM30D, and IRF1 (interferon regulatory factor 1) proteins were 87, 81, 94, 79, and 99% identical to their counterpart *P. maniculatus* sequences, respectively. The TRIM30D sequence from *P. leucopus* was the one most closely related to TRIM30D sequences from the other species at 92% sequence identity. The MAVS, IFNAR1, STAT1, and IRF1 sequences were divergent (55–89% sequence identity). Thus, these results suggested that the *P. maniculatus* genome was like that of *P. leucopus* as expected and that it could be used to infer our RNA-Seq data.

### Analysis of significantly differentially expressed genes during early POWV infection.

**Fig. 1:**
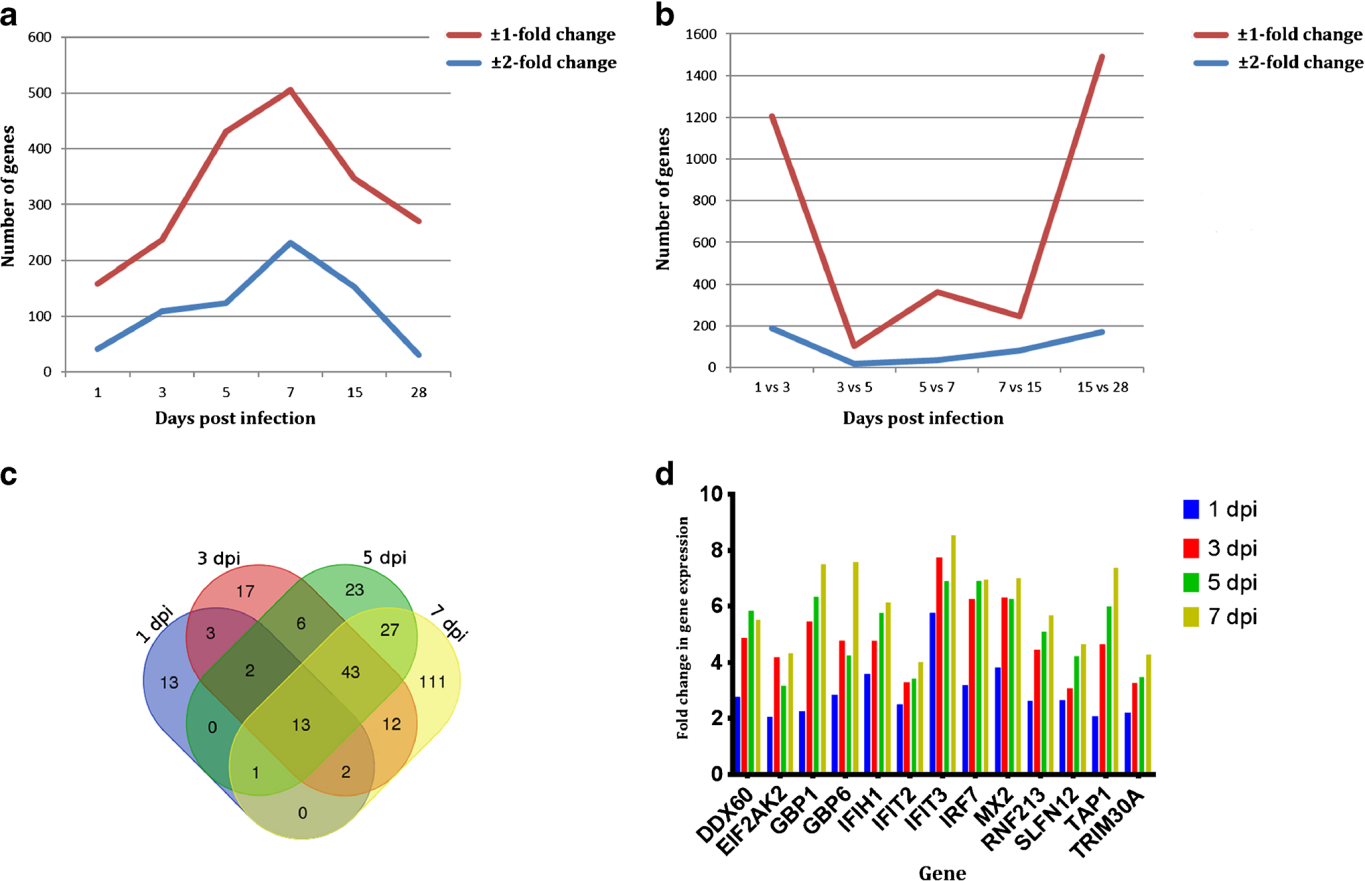
Overview of the differential gene expression at different time points following POWV challenge in *P. leucopus* mice. **a** The trend in the number of genes that were significantly differentially expressed in *P. leucopus* mouse brains in comparison to uninfected controls. **b** Comparison of the gene expression profiles of POWV-infected mouse brains. **c** Venn diagram showing the number of genes (± 2-fold change in expression) which were common or different during POWV infection from 1 to 7 dpi. **d** Differential expression of the 13 genes that was common to the 1-, 3-, 5-, and 7-dpi time points. The expression levels were relative to the mock-infected samples

In our previous study, we observed mild encephalitis in *P. leucopus* mouse brains from 5 to 15 dpi (Mlera et al. 2017). Defining early POWV infection as the period from 1 to 7 dpi following intracranial (ic) inoculation, we determined the number of significantly differentially expressed genes in comparison to mock-infected animals. Significance was called using a false discovery rate or *q* value of 0.05- and ± 2-fold change in gene expression. One day after ic infection, 39 genes had increased in expression level, whereas 3 were downregulated (Fig. 1a, Table 1). *HIST1H2BA* (histone cluster 1 H2B family member A), *HIST1H4A* (histone cluster 1 H4 family member A), and *ISCU* (iron-sulfur cluster assembly enzyme) were the downregulated genes.

**Table 1:** Summary of the number of genes that were significantly differentially expressed in *P. leucopus* mouse brains following inoculation with POWV in comparison to the mock-inoculated brains

	Days post infection					
	1	3	5	7	15	28
Number of genes at ± 2-fold change^a^	42	109	124	232	153	31
Number of genes at ± 1-fold change^b^	159	237	431	505	347	270
Total significant genes	379	371	715	787	451	2031

^a^The ± 2-fold change denotes gene expression levels higher than 2-fold upregulation or lower than − 2-fold downregulation

^b^The ± 1-fold change denotes gene expression levels higher than 1 (including a 2-fold increase) or lower than − 1 (including − 2-fold downregulation)

At 3 dpi, only 2 of 109 genes with significant changes in expression were downregulated, and these were *HIST1H4A* and *COX5B* (cytochrome C oxidase subunit 5B). The number of genes with significant changes in expression peaked at 7 dpi, but only four were downregulated, *CRB1* (crumbs 1), *ADAMTS19* (ADAM metallopeptidase with thrombospondin type 1 motif 19), *FRMD7* (FERM domain containing 7), and *PRDX6* (peroxiredoxin 6). Thus, these data indicated a progressive increase in the number of genes whose expression was induced from 1 to 7 dpi.

We evaluated the genes that were commonly expressed at all time points from 1 to 7 dpi and depicted the results in a Venn diagram (Fig. 1c). Thirteen genes were common to all early POWV infection time points. These genes fell into two main families: (a) genes involved in RNA sensing, i.e., *DDX60* (DExD/H-box helicase 60) and *EIF2AK2* (eukaryotic translation initiation factor 2 alpha kinase 2), and (b) genes with known antiviral properties, i.e., *IFIT2* (interferon-induced protein with tetratricopeptide repeats 2), *IFIT3*, *TRIM30A* (tripartite motif containing 30A, a *TRIM5α* homolog), *MX2* (myxovirus resistance protein 2), *IFIH1* (interferon induced with helicase C domain 1), *GBP1* (guanylate-binding protein 1), *GBP4*, and *GBP6*. Other genes common to the early time points included *SFLN12* (schlafen 12), *RNF213* (ring finger protein 213), and *IRF7*. Expression levels of some of these genes at each time point are shown in Fig. 1d, and the clear general trend was an increase in fold change over time.

### Analysis of significant gene expression at 15 dpi

We set 15 dpi as the midpoint between the early infection period and the experimental endpoint (28 dpi). This was the time at which encephalitis was more pronounced as shown by histopathological examination of brain tissue sections (Mlera et al. 2017). At this time point, 153 genes were significantly upregulated (Fig. 1a, Table 1), and none were downregulated. Several RNA-sensing dead-box helicases were upregulated at 15 dpi; *DDX60* (3.2-fold) and *DHX58* (DExH-box helicase 58, 2.6-fold). In addition, six genes (*SLFN12*, *MX2*, *TRIM30A*, *GBP1*, *GBP6*, and *IFIH1*) were common to 15 dpi and the early infection period. In total, 49 of the 153 genes that were significantly upregulated at 15 dpi were unique to this time point, and the overall level of gene activation was reduced.

### Analysis of significant gene expression at 28 dpi

We previously showed that POWV RNA persisted in *P. leucopus* mouse brains at an average of 778 copies/μg of total RNA at 28 dpi following ic inoculation, corresponding to ~ 0.004 genomes/cell (Mlera et al. 2017). Thus, we examined the differential gene expression at 28 dpi to gain insight into the *P. leucopus* mouse brain response to this residual viral RNA. The results showed that the total number of RNA-sensing helicases that were significantly differentially expressed (*q* < 0.05) was higher than the earlier time points, but the range of expression levels was lower than + 2-fold and higher than − 2-fold change. The DExD-box helicases whose expression levels were up (> 0 and + 2<) were *DDX17*, *DDX18*, *DDX26*, *DDX46*, *DDX52*, and *DDX58*, and the fold change was in the range 0.42 to 0.67. In contrast, other RNA-sensing helicases, such as *DDX23*, *DDX27*, *DDX49*, and *DDX54*, were downregulated with the fold change in the range − 0.70 to − 0.46. These results suggested that the small amount of viral RNA still present was sufficient to impact pathogen recognition receptor sensing.

Thirty-one genes were significantly differentially upregulated at ± 2-fold change at 28 dpi (Table 1, Fig. 1), whereas only two genes, *FOXO6* (forkhead box O6) and *HIST1H4A*, were downregulated. Both *FOXO6* and *HIST1H4A* are involved in transcription regulation. Only *MX2*, *CP* (ceruloplasmin), and *H2-Ea* (H-2 class II histocompatibility antigen, E-D alpha chain) in the upregulated gene set overlapped with any of the earlier time points from 1 to 15 dpi.

Compared to earlier time points, the most dramatic difference in the number of genes was observed between 15 and 28 dpi where 170 genes were different at ± 2-fold (Fig. 1b, Table 2). Thus, these results indicated that the transcriptome profile of *P. leucopus* mouse brains at 28 dpi was dramatically different from early infection time points.

**Table 2:** Comparison of significant gene expression between the POWV-inoculated mouse brains over time

	Time points compared				
	1 vs 3 dpi	3 vs 5 dpi	5 vs 7 dpi	7 vs 15 dpi	15 vs 28 dpi
Number of genes at ± 2-fold change	189	17	36	81	170
Number of genes at ± 1-fold change	1207	102	364	246	1490
Total significant genes	2988	116	411	273	4299

### Ingenuity Pathway Analysis of the *P. leucopus* brain response to POWV

Using a systems biology approach, we employed the IPA software to interrogate the canonical pathways responding to POWV infection in the brains of *P. leucopus* mice. We present the top 5 canonical pathways (based on *p* value) that were part of the POWV response in the brains as presented in Table 3. One pathway hit during early infection time points was “The role of pathogen recognition receptors (PRRs) in the recognition of bacteria and viruses” (Table 3). The PRRs, in turn, induce the interferon signaling system. Thus, it was important to note that *interferon (IFN) signaling* was a pathway that appeared at all time points, except at 28 dpi. From 3 to 15 dpi, *IFN signaling* was at the top (*p* value) of all the pathway hits, suggesting that this pathway was an important and integral part of the *P. leucopus* mouse brain response to POWV until very late after infection.

**Table 3:** Summary of top 5 canonical pathways inferred from the IPA over the course of POWV infection in the brains of *P. leucopus* mice

Time (dpi)	Canonical pathway	*p* value
1	Role of PRRs in the recognition of bacteria and viruses	3.47*E*−05
	Activation of IRF	8.19*E*−05
	Interferon signaling	1.1*E*−03
	Role of RIG-like receptors in antiviral activity	1.57*E*−03
	Calcium transport	1.36*E*−02
3	Interferon signaling	8.29*E*−15
	Activation of IRF by cystosolic PRRS	8.09*E*−11
	Antigen presentation pathway	9.42*E*−11
	Role of PRRs in the recognition of bacteria and viruses	8.53*E*−07
	Crosstalk between dendritic and natural killer cells	1.07*E*−06
5	Interferon signaling	2.73*E*−16
	Role of PRRs in the recognition of bacteria and viruses	1.53*E*−11
	Activation of IRF by cystosolic PRRs	2.20*E*−10
	Dendritic cell maturation	8.79*E*−09
	Antigen presentation pathway	1.24*E*−08
7	Interferon signaling	7.12*E*−15
	Role of PRRs in the recognition of bacteria and viruses	5.65*E*−14
	TREM1 signaling	7.42*E*−14
	Antigen presentation pathway	6.73*E*−13
	Dendritic cell maturation	6.26*E*−12
15	Interferon signaling	3.31*E*−13
	Antigen presentation pathway	5.67*E*−13
	Complement system	1.3*E*−09
	T helper cell differentiation	7.57*E*−09
	Dendritic cell maturation	9.96*E*−09
28	Acute-phase response signaling	7.22*E*−18
	LXR/RXR activation	3.99*E*−13
	FXR/RXR activation	5.56*E*−13
	Coagulation system	2.97*E*−10
	Extrinsic prothrombin activation pathway	1.88*E*−09

The IPA also enables inference of upstream regulators, i.e., molecules likely to be responsible for the observed phenotype or activation/inhibition of other molecules. This analysis suggested that TRIM24 (transcription intermediary factor 1α) was inhibited at every time point from 1 to 15 dpi (Table 4). Although TRIM24 inhibition was not in the top 5 at 7 dpi, it was in the top 10 upstream regulators with a *p* value of 1.18*E*−40 and a *z*-score of − 5.509. This result was interesting because TRIM24 inhibits many IFN-induced genes and may also regulate the IFN response.

**Table 4:** Analysis of the top (*p* value) upstream regulators of the *P. leucopus* mouse brain transcriptome profiles

Time (dpi)	Upstream regulator	Predicted activation	Activation *z*-score	*p* value of overlap
1	TRIM24	Inhibited	− 3.0	5.59*E*−15
	IFN-α2	Activated	3.251	6.81*E*−15
	IRF7	Activated	3.107	1.03*E*−14
	ACKR2	Inhibited	− 2.646	4.10*E*−14
	IRF3	Activated	3.056	5.92*E*−14
3	IFN-γ	Activated	7.020	9.28*E*−47
	IFN-α2	Activated	5.625	5.17*E*−44
	IRF7	Activated	5.348	1.79*E*−43
	TRIM24	Inhibited	− 5.042	2.44*E*−42
	IFNAR	Activated	4.577	2.05*E*−41
5	IFN-γ	Activated	7.042	4.06*E*−49
	TRIM24	Inhibited	− 5.140	1.95*E*−42
	IRF7	Activated	5.348	4.06*E*−41
	IFNAR	Activated	4.940	1.58*E*−39
	IFN-α2	Activated	5.442	6.85*E*−38
7	IFN-γ	Activated	9.425	1.02*E*−73
	IFNA2	Activated	6.848	1.41*E*−57
	IFNAR	Activated	5.670	6.93*E*−54
	IFN-α	Activated	6.543	1.13*E*−50
	LPS	Activated	7.964	1.27*E*−50
15	IFN-γ	Activated	7.045	8.46*E*−49
	IFNAR	Activated	4.247	4.99*E*−31
	TRIM24	Inhibited	− 4.626	9.65*E*−30
	IRF7	Activated	4.773	1.74*E*−27
	IFN-α	Activated	4.703	2.81*E*−26
28	HNF1A	Activated	2.804	2.94*E*−13
	Nitrofurantoin	Inhibited	− 2.630	5.30*E*−13
	Carbon tetrachloride	–	0.577	2.25*E*−11
	IL-6	Activated	2.098	5.57*E*−11
	Methapyrilene	Inhibited	− 2.449	1.01*E*−10

The other upstream regulators that were in the top 5 (*z*-score and *p* value) at each time point included IFNAR, IFN-α2, IFN-γ, and IRF7. IFNAR and IFN-γ were in the top 5 at 3 to 15 dpi, whereas IFN-α2 ranked high at 1 to 7 dpi (Table 4).

The IPA of the data collected at 28 dpi showed that the top 5 canonical pathways at that point included *acute-phase response signaling* (*p* = 7.218*E*−18), *activation of the liver X receptor with retinoic X receptor* (LXR/RXR; *p* = 3.99*E*−13), and *farnesoid X receptor with RXR* (FXR/RXR; *p* = 5.56*E*−13). These signaling pathways were characterized by the upregulation of genes encoding for SERPINA1 (alpha-1-antitrypsin precursor); apolipoproteins A1, A2, and H; as well as fibrinogen α, β, and γ chains. LXR/RXR and FXR/RXR systems are involved in cholesterol homeostasis as well as counteracting inflammation. Although we did not observe any obvious signs of inflammation at 28 dpi by histopathological examination of brain sections (Mlera et al. 2017), the transcriptome profile also suggested that the acute-phase response signaling was activated (Table 3) and that there was activation of upstream regulators involved in inflammation, such as IL-6 (Table 4). Thus, there was a clear residual molecular footprint of inflammation in *P. leucopus* mouse brains at 28 dpi.

### Analysis of the expression of IFN-stimulated genes

**Fig. 2:**
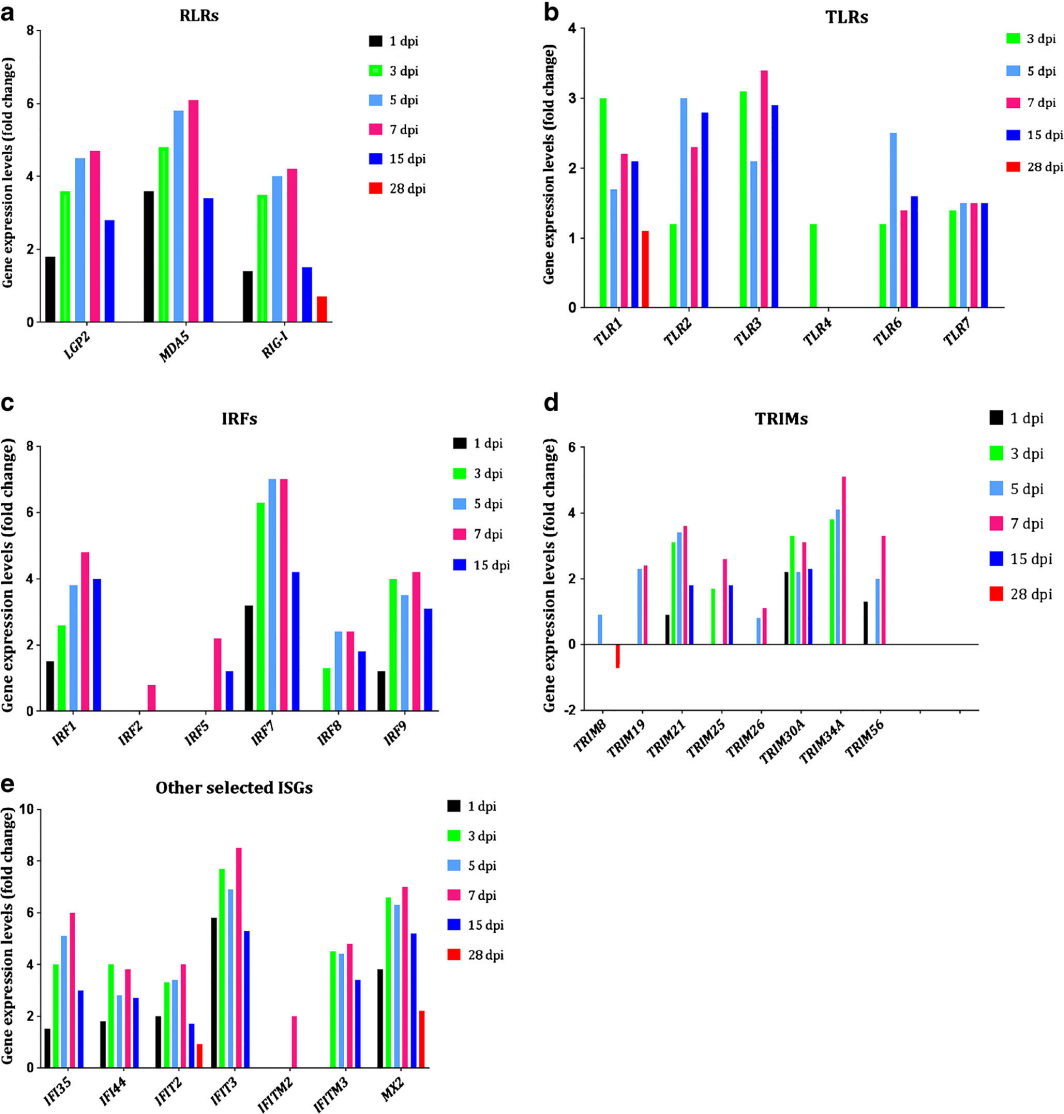
Analysis of the expression level of selected genes representing RIG-like receptors (**a**), TLRs (**b**), IRFs (**c**), and ISGs (**d**, **e**). The expression levels are relative to the mock-infected *P. leucopus* brain samples

Initial analyses with IPA indicated that a robust IFN signaling response was a prominent feature of the *P. leucopus* mouse brain response to POWV infection (Table 3). IFN signaling is activated by host cell viral RNA sensors, and our *P. leucopus* mouse brain transcriptome sequencing results showed the upregulation of RIG-like receptors RIG-I, MDA5, and LGP2 (Fig. 2a). In addition, genes encoding Toll-like receptors TLR1–TLR4, TLR6, and TLR7 were also upregulated (Fig. 2b), suggesting that POWV-associated molecular patterns were also detected by these membrane-bound pathogen recognition receptors.

**Fig. 3:**
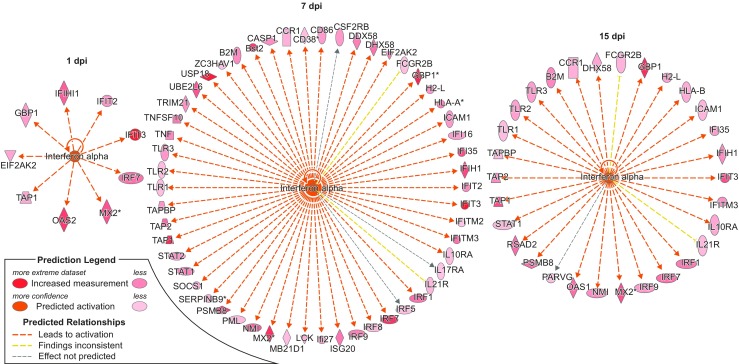
IPA depicting activation of IFN-α and its effect on the upregulation of other genes at 1, 7, and 15 dpi. Only 9 genes were upregulated at 1 dpi, followed by a dramatic increase to 54 genes at 7 dpi, and the number declined to 30 by 15 dpi. The red color in the genes indicates upregulation, and the intensity of the color represents gene expression levels, i.e., the more intense, the more upregulated

Following IFN-α secretion, a barrage of IFN-stimulated genes (ISGs) was expressed in an incremental fashion over time as expected (Figs. 2d, e, and 3). Some of the genes that code for the ISGs include those that code for tripartite motif (TRIM) containing proteins, and we noted that *P. leucopus* mice responded to POWV by upregulating the following array of TRIMs: *TRIM8*, *TRIM19*, *TRIM21*, *TRIM25*, *TRIM26*, *TRIM30A* (homolog of human *TRIM5α*), *TRIM34A* (*TRIM6* paralog), and *TRIM56*. *TRIM21* and *TRIM30A* were the only genes that were upregulated at 1 to 15 dpi (Fig. 2d), and the trend of gene expression was incremental over each of these time points, peaking at 7 dpi and then progressively declining at 15 and 28 dpi. *TRIM34A* gene expression was the most upregulated at 3, 5, and 7 dpi. The upregulation of various TRIM-encoding genes during POWV infection suggests that these may be playing a collective or synergistic role in virus restriction.

Other ISG-encoding genes of interest were *IFIT3* and *MX2*, which are known to have antiviral properties and were expressed to elevated levels by 7 dpi (Fig. 2e). *IFIT3* was expressed to the highest level of 8.5-fold at 7 dpi, whereas the highest expression level of *MX2* was 7-fold, and it was upregulated at all time points from 1 to 28 dpi (Fig. 2e). The upregulated expression of *MX2* by 28 dpi suggested that the antiviral system was still active and that MX2 might also play a crucial role in the response against POWV in *P. leucopus* mouse brains.

IFN secretion is controlled by IRFs, and interrogation of the RNA-Seq results showed that *IRF1*, *IRF7*, and *IRF9* were incrementally upregulated from 1 dpi and the expression levels peaked at 7 dpi, before slightly declining at 15 dpi (Fig. 2c). *IRF7* gene expression was the most upregulated at each of these time points. *IRF2* was only significantly upregulated at 7 dpi, but the expression level was < 2-fold. *IRF5* was significantly upregulated at 7 and 15 dpi only, whereas *IRF8* was upregulated from 3 to 15 dpi, but at relatively lower expression levels.

### Validation of RNA-Seq data

**Fig. 4:**
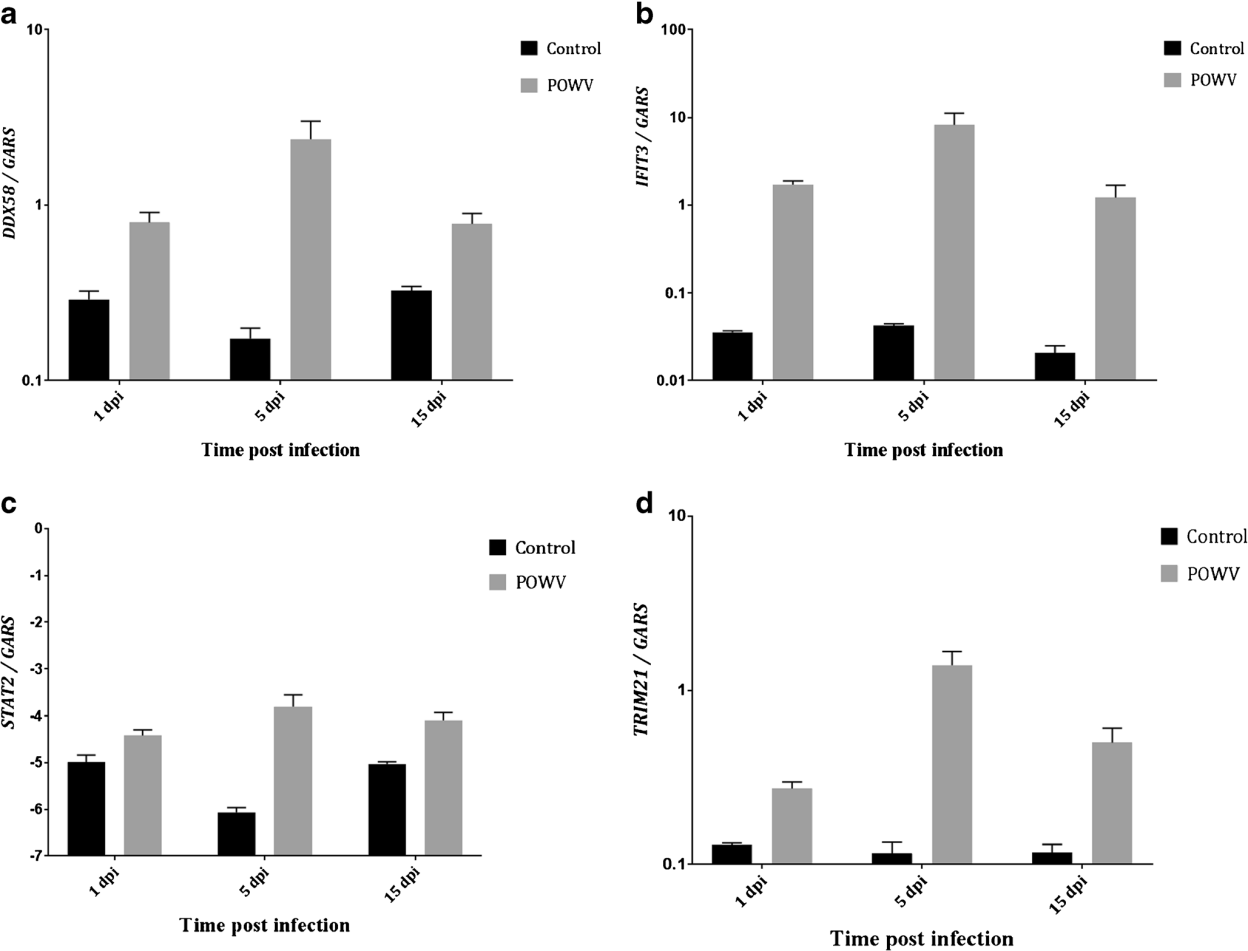
Validation of next-generation sequencing (NGS) data with qRT-PCR. The expression levels for each gene analyzed were relative to the expression levels of the *GARS* gene, and the results corroborated with the gene expression levels obtained by NGS (Table 5). The values depict the average gene expression levels for three mouse brains, and the error bars indicate the standard error of the mean (SEM)

We used qRT-PCR to validate the RNA-Seq data, and for this purpose, we selected genes associated with innate immune responses, i.e., *DDX58*, *IFIT3*, *STAT2*, and *TRIM21*. Corroboration assays were performed using cDNA samples from 1-, 5-, and 15-dpi time points. The gene expression levels for *DDX58*, *IFIT3*, *STAT2*, and *TRIM21* are shown in Fig. 4, and the Spearman correlation coefficients are listed in Table 5. The qRT-PCR data correlated well (*p* values in Table 5) with the RNA-Seq data; thus, we concluded that the RNA-Seq data could be used for biological inference (Table 6).

**Table 5:** qRT-PCR validation of RNA-Seq data: Spearman correlation coefficients and *p* values

Gene	Spearman correlation coefficient	*p* value
*STAT2*	0.847	9.457*E*−06
*IFIT3*	0.859	4.942*E*−06
*DDX58*	0.875	2.010*E*−06
*TRIM21*	0.821	2.980*E*−05

**Table 6:** Primers used for q-RT-PCR validation of RNA-Seq data

Gene	Accession	Oligo type	5′–3′ sequence
*STAT2*	NM_019963	Forward	CCGAAGTCCCAAATTAAGCC
		Reverse	AGCGAATCACTCAAAGCAGA
		5′ 6-FAM/ZEN/3IABkFQ 3′	CCGGAAGCCTGGTAAGTCTGAATTCC
*IFIT3*	NM_010501	Forward	CTTCAGCTGTGGAAGGATCG
		Reverse	CACACCCAGCTTTTCCCA
		5′ 6-FAM/ZEN/3IABkFQ 3′	CACCATCATGAGTGAGGTCAACCGG
*DDX58*	NM_172689	Forward	CCTTGTTGTTCTTCTCAGCCT
		Reverse	CCACCTACATCCTCAGCTACA
		5′ 6-FAM/ZEN/3IABkFQ 3′	TGTACTGCACCTCCTCATCCTCGA
*TRIM21*	NM_009277	Forward	CTTTGATCCTTCTCCAGCCT
		Reverse	CACGATGCAAAGAACAGACTG
		5′ 6-FAM/ZEN/3IABkFQ 3′	ATTCACGCAGAGTTCGCACTTCAGA
*GARS*	NM_180678	Forward	GTCAGCACATCCAACAATCTC
		Reverse	CTCCTGATAAACTCCGCTTCC
		5′ HEX/ZEN/3IABkFQ 3′	CCGAGTCCAAAACGTCCTATGGCT

## Discussion

Powassan virus causes life-threatening encephalitis in humans. This virus is transmitted by hard-bodied *Ixodes* tick species, and it seems very likely that human infection with POWV is on the increase (Ebel 2010; Piantadosi et al. 2015). The *Ixodes* ticks obtain blood meals from small-to-medium-sized mammals, which include *P. leucopus* mice, but very little is known regarding the relationship between these animals and POWV. To improve knowledge in this subject matter, we developed a *P. leucopus* mouse model of POWV infection in which the virus does not cause obvious clinical signs of disease, but intracranial inoculation resulted in mild inflammation. Evidence of virus replication was restricted in time and space mainly to the olfactory bulb (Mlera et al. 2017). To further characterize the *P. leucopus* mouse brain response to POWV, we used RNA-Seq to profile the transcriptome of intracranially inoculated *P. leucopus* mice and compared it to mock-inoculated mice.

Results of our RNA-Seq study showed that early after infection, there was a progressive increase in the number of genes that were significantly differentially expressed. The total number of significantly differentially expressed genes peaked to 232 (± 2-fold change in expression levels) at 7 dpi. By 28 dpi, the number of significantly differentially expressed genes had declined to only 28, suggesting a well-controlled transcriptome response to POWV. These results were consistent with our previous observations that infectious POWV was only detectable by culture in the first 7 days of infection (Mlera et al. 2017). It is also interesting to note that intraperitoneally or intracranially inoculated 4-week-old BALB/c mice succumb to POWV disease within the first week of infection, suggesting that there are unique factors of the *P. leucopus* mouse response that are expressed early and critical for restricting POWV without extensive pathology in the host.

The emergent theme in the transcriptome profile was that *P. leucopus* mouse brains mounted a robust and well-controlled interferon response (Figs. 2 and 3, Tables 3 and 4) that was effective in controlling the spread of infection. This is exemplified by a change in the landscape of IFN-α signaling, which starts with just nine upregulated IFN-stimulated genes at 1 dpi, followed by a dramatic increase to 54 upregulated ISGs by 7 dpi (Fig. 3). Indeed, the IFN response is a well-known potent antiviral host response system, which leads to the transcription of hundreds of virus-incapacitating ISGs (Raftery and Stevenson 2017; Wang et al. 2017). Thus, it was not surprising to see the increase in the genes affected by IFN-α. The IFN response is also activated in the brains of Swiss Webster mice challenged with the mosquito-borne West Nile virus (WNV) or Japanese encephalitis virus, and in the brains of C57BL/6 mice challenged with WNV (Clarke et al. 2014; Kumar et al. 2016). Importantly, unlike *P. leucopus* mice infected with POWV, the C57BL/6 and Swiss Webster mice develop widespread infection and neurological symptoms and succumb from disease. A recent report has shown that IFN signaling is associated with restriction of POWV replication in vitro in adult and embryonic *P. leucopus* fibroblasts, in comparison to *Mus musculus* fibroblast cells (Izuogu et al. 2017). In this report, the authors found that both *P. leucopus* and *M. musculus* fibroblast cells secrete IFN upon challenge with the tick-borne Langat virus and that knockdown of STAT1 or IFNAR1 increased viral replication in *P. leucopus* fibroblasts (Izuogu et al. 2017). Although we were not able perform direct *P. leucopus* and *M. musculus* comparisons in vivo, results from the WNV studies also suggest that the IFN response is, in and of itself, insufficient to control virus replication. This implies that there may be additional factors that restrict POWV replication and disease induction not typically associated with the control/regulation of IFN signaling or, perhaps indeed, even novel factors completely unrelated to the classical IFN antiviral system. We are currently pursuing studies aimed at decoding the ~ 40% RNA-Seq reads that we were unable to map to a reference genome as a way of deciphering these factors.

The specific ISGs common to early POWV replication included *DDX60*, *GBP1*, *GBP4*, *GBP6*, and *MX2*; the products of all these genes are antiviral. DDX60 mediates its antiviral properties by binding to RIG-I to promote RIG-I-like signaling (Miyashita et al. 2011). The guanylate-binding proteins (GBPs) are IFN inducible, and GBP1 has been shown to be upregulated with an inhibitory effect during dengue virus infection (Pan et al. 2012). Our results showed that in addition to GBP1, GBP4 and GBP6 are also upregulated during POWV infection. Similar results have been reported for WNV infection in Swiss Webster mice (Clarke et al. 2014).

The use of IPA enables us to observe that TRIM24 was inhibited over the entire course of POWV infection. This was an interesting finding because TRIM24 is proposed to be a negative regulator of IFN signal transducers and activators through retinoic acid receptor alpha (RARA). In TRIM24 knockout cells, many ISGs, genes such as *IFIT2*, *IFIT3*, and *IFIH1*, were found to be TRIM24-dependent (Tisserand et al. 2011). POWV does not replicate to high titers in *P. leucopus* mouse brains, and the suppression of TRIM24 suggests that this molecule may be a crucial factor in controlling the IFN response and, subsequently, POWV replication. Thus, further experiments to explore the role of TRIM24 in POWV replication or host response are warranted.

In contrast to TRIM24, genes that code for several other TRIMs were upregulated in expression and these included *TRIM19*, *TRIM21*, *TRIM25*, *TRIM30A*, and *TRIM34A* (Fig. 2d). There is a cornucopia of TRIMs, and they have varied functions, including cell proliferation, differentiation, as well as antiviral activity (Nisole et al. 2005; Rajsbaum et al. 2008). TRIM79α is rodent-specific and was shown to inhibit the tick-borne Langat virus by targeting the RNA-dependent RNA polymerase (NS5) for lysosomal degradation (Taylor et al. 2011). The precise role of the TRIMs we identified is unclear, and it will be interesting to delineate the specific role played by each one of the TRIMs identified in our study with POWV. In vitro modeling with *P. leucopus* cells may prove a more tractable system for elucidating these complicated networks, and we are currently undertaking this work.

The 28-dpi differential gene expression profile was different from the rest of the time points we analyzed. This was not unexpected, considering that the times were far apart and that the persistent POWV RNA was no longer associated with any infectious POWV (Mlera et al. 2017). However, it was interesting that we did not observe any signs of inflammation at 28 dpi by histology (Mlera et al. 2017), but the IPA suggested that some elements of the acute-phase response signaling remained active, indicative of inflammation. In addition, the IPA of the 28-dpi gene set indicated the activation of the LXR/RXR and FXR/RXR systems (Table 3). LXR is a heterodimeric transcription factor involved in cholesterol metabolism, and it also has anti-inflammatory activity (Tall and Yvan-Charvet 2015). LXR has been reported to have antiviral activity for several viruses. For example, stimulation of the LXR with LXR agonists resulted in potent inhibition of HIV replication in a humanized mouse model (Ramezani et al. 2015). Nakajima et al. showed that neoechinulin B inhibits LXR and subsequently inhibits HCV replication because it reduced double-membrane vesicles in which HCV replication occurs (Bocchetta et al. 2014; Nakajima et al. 2016; Zeng et al. 2012). Langat virus and the mosquito-borne Zika virus also cause an expansion of membrane-bound vesicles in the endoplasmic reticulum (Offerdahl et al. 2012, 2017), suggesting that blocking LXR could inhibit POWV replication, a hypothesis that needs further study. In contrast, the LXR/RXR genes seem to have a pro-viral effect in the case of Coxsackie virus B3 (CVB3), and it does not reduce cardiac inflammation in vivo, predisposing mice to mortality upon infection (Papageorgiou et al. 2015). At present, we are uncertain of the role of LXR/RXR and FXR/RXR activation during POWV infection and this merits further study.

In summary, we determined the brain transcriptome profile of *P. leucopus* mice following intracranial inoculation with POWV and compared the results to those of mock-inoculated animals. There was an increase in the number of genes that were significantly differentially expressed from 1 to 7 dpi, followed by a decline at 15 and 28 dpi. The IPA of the genes at 1 to 15 dpi indicates that *P. leucopus* mice infected with POWV mount a robust IFN response, which is characterized by the upregulation of many antiviral genes. Some of the induced genes include *GBP1*, *GBP4*, *GBP6*, several *TRIMs*, and *MX2*. As mentioned earlier, further studies and development of in vitro systems will prove valuable to delineate restriction factors of POWV, and these results will be useful for studies aimed at the development of POWV antiviral therapies.
